# Advancements and Future Perspectives of Human Papillomavirus (HPV) Vaccination in Latin America: Insights from Recent Decades

**DOI:** 10.3390/healthcare13192502

**Published:** 2025-10-02

**Authors:** Marcela Bonalumi dos Santos, Martina Parenza Arenhardt, Giovanna Vieira Giannecchini, Larissa Müller Gomes, Jessé Lopes da Silva, Diocesio Alves Pinto de Andrade, Andréia Cristina de Melo

**Affiliations:** 1 Department of Medical Oncology, Oncoclinicas&Co, São Paulo 01304-001, Brazil; giovanna.giannecchini@medicos.oncoclinicas.com (G.V.G.); larissa.gomes@medicos.oncoclinicas.com (L.M.G.); jesse.silva@medicos.oncoclinicas.com (J.L.d.S.); diocesio.andrade@medicos.oncoclinicas.com (D.A.P.d.A.); andreia.melo@medicos.oncoclinicas.com (A.C.d.M.); 2 Irineu Boff Family Oncology Center, Nora Teixeira Hospital, Santa Casa de Porto Alegre, Porto Alegre 90020-090, Brazil; martina.arenhardt@santacasa.org.br; 3 Division of Clinical Research and Technological Development, Brazilian National Cancer Institute, Rio de Janeiro 20231-050, Brazil

**Keywords:** HPV, vaccine, cervical cancer, cancer prevention, immunization, Latin America

## Abstract

Despite being a preventable disease, cervical cancer remains a significant public health concern in low- and middle-income countries, including those in Latin America (LATAM), where mortality rates are nearly three times higher than in North America. HPV vaccination represents one of the most important strategies for cervical cancer elimination; however, uptake in these regions has been consistently suboptimal. The aim of this review is to analyze the current status of HPV vaccination programs across LATAM, examines the underlying challenges, and proposes strategies to enhance vaccine coverage. Multiple obstacles to widespread vaccine adoption persist, including limited awareness, cultural stigma, and regional disparities in healthcare access, often driven by socioeconomic and infrastructural limitations. Addressing these challenges through multifaceted interventions—such as school-based vaccination programs, healthcare provider engagement, digital dissemination, simplified dosing schedules, and supportive policy measures—is essential to effectively improve vaccination rates and reduce disease burden.

## 1. Introduction

Although the incidence of cervical cancer (CC) is low in high-income countries (HICs), it remains widespread in low- and middle-income countries (LMICs), ranking as the eighth most common tumor worldwide and accounting for nearly 350,000 deaths annually [1]. Given that infection by the *Human Papillomavirus* (HPV) is a key factor in the development of most CC, this tumor’s pathophysiology helps explain the significant regional differences in incidence rates observed globally [2].

HPV is transmitted through sexual contact and is associated with anogenital and oropharyngeal diseases—including cervical, anal, penile, vulvar, vaginal, and oropharyngeal cancers—underscoring its substantial public health burden. Approximately 75% of CC cases worldwide are related to high-risk HPV genotypes 16 and 18. An additional 20% of cases may be linked to 5 other genotypes described as follows: HPV-31, HPV-33, HPV-45, HPV-52 and HPV-58 [3]. HPV vaccines play a central role in the prevention of HPV-related cancers. Currently, three types are available: bivalent (Cervarix), tetravalent (Gardasil), and nonavalent (Gardasil-9) [4,5,6,7].

The World Health Organization (WHO) has proposed a comprehensive strategy for the elimination of CC, based on three key principles to be adopted by countries worldwide, the well-known “90-70-90 program”: first, 90% of girls should be fully vaccinated with the HPV vaccine by age 15; second, 70% of women should undergo screening with a high-performance test by ages 35 and 45; and third, 90% of women diagnosed with pre-cancer or CC should receive appropriate treatment [8].

While the implementation of the “90-70-90 program” may be feasible in HICs, its execution in LMICs often faces significant challenges [9,10]. Therefore, this review aims to examine the current landscape of HPV vaccination in Latin America (LATAM) and to propose strategies to enhance vaccination coverage across the region.

## 2. Epidemiology of HPV-Related Cancers in Latin America

Across LATAM, over 1.5 million new cancer cases are expected each year according to data from GLOBOCAN 2022, with prostate cancer, colorectal cancer, and lung cancer being the three most prevalent in males and breast cancer, colorectal cancer, and CC being the most prevalent in females. Considering tumors potentially related to HPV infection (CC, vulvar, vaginal, anal, penile, oropharynx), the estimated annual incidence exceeds 83.000 cases per year, with CC alone accounting for approximately 630,000 [1]. In 2017, de Martel and colleagues estimated that 78,000 cases of cancer in LATAM were associated with HPV infection, including 69,000 cases of CC [11].

**Figure 1:**
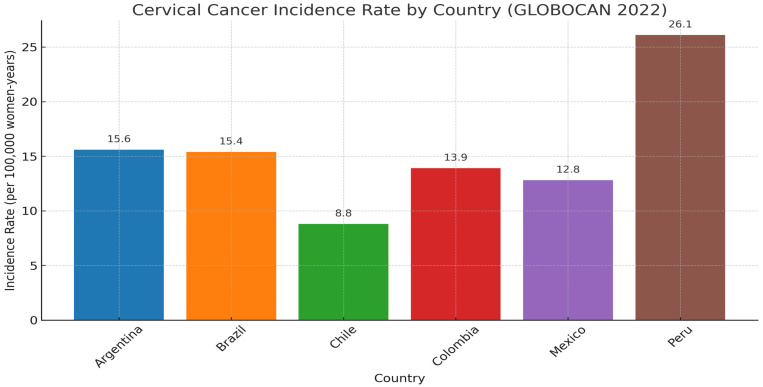
Cervical Cancer Incidence Rate (per 100,000 women-years) [1].

The two countries with the highest rates of CC diagnosis in LATAM are Bolivia (36.6 cases per 100.000 women-years) and Paraguay (34.1 cases per 100.000 women-years). Conversely, between 1988 and 2017, a marked decline in CC incidence was observed in countries such as Ecuador, Chile, Brazil, Colombia, and Costa Rica [1]. Figure 1 presents a compilation of current CC incidence rates for several LATAM countries.

Analyzing cancers attributed to some type of infection in 2018, just over 800,000 cases were directly related to *Helicobacter pylori* and 690,000 cases were directly related to HPV infection, especially CC [12]. Using age-standardized incidence rates (ASIR) of HPV-attributable cancers in LATAM, incidence was estimated at 8.0 cases per 100,000 person-years in the Caribbean and Central America, and 9.6 cases per 100,000 person-years in South America [13].

## 3. Vaccine Types, Coverage and Efficacy

The immune response provided by HPV vaccines is significantly more effective than that of the natural virus, underscoring the importance of prophylactic vaccination as a central strategy for disease prevention due to the critical role of host immunity in viral clearance. Currently, licensed HPV vaccines are formulated using virus-like particles (VLPs) which are non-infectious and non-oncogenic, providing a safer alternative to HPV-attenuated vaccines [14]. Three HPV vaccines—the nine-valent (Gardasil 9, 9vHPV), quadrivalent (Gardasil, 4vHPV), and bivalent (Cervarix, 2vHPV)—are available in many countries, targeting various HPV strains [15]. Firstly, Cervarix protects against HPV types 16 and 18, which are responsible for approximately 70% of CCs [16]. In addition, Gardasil targets these same HPV types and also addresses types 6 and 11, which account for 90% of genital wart cases [17]. Furthermore, Gardasil 9 provides extensive protection against HPV types 6, 11, 16, 18, 31, 33, 45, 53, and 58, covering an additional 20% of CC cases and offering protection for nearly 90% of this type of cancer [18].

The real-world impact of HPV vaccination is substantial, with reported reductions of approximately 90% for HPV types 6, 11, 16, and 18 infections, as well as significant reductions in genital warts and cervical abnormalities, yielding estimated vaccine efficacy ranging from 83% to 96.1% [19]. Additionally, vaccine effectiveness depends on multiple factors, including age at immunization, immunosuppressive status, geographical variations in HPV type distribution, and—critically—adherence to the vaccination schedule [20,21,22].

## 4. Vaccine Implementation in LATAM—Background, National Programs and Countries’ Target Populations

The WHO made an announcement endorsing the routine inclusion of HPV vaccination for females in national immunization programs in April 2009. This recommendation is based on key criteria: prioritizing CC and HPV-related diseases as public health concerns, ensuring programmatic feasibility for vaccine introduction, securing sustainable funding, and evaluating the cost-effectiveness of vaccination strategies for each country or region [23]. WHO asserts that “HPV vaccines should be introduced as part of a coordinated strategy to prevent CC” and warns that funding for these vaccines should not compromise resources for other effective CC screening programs [24].

In LATAM, the integration of HPV vaccination into immunization programs began in Panama and Mexico. In 2008, Panama incorporated a bivalent HPV vaccine into its national immunization program, targeting girls aged 10, and made it available through adolescent health services in clinics and schools. By 2009, one-dose coverage among 10-year-old girls reached 89%, while completion of the three-dose series was only 46% [25]. Similarly, in Mexico, the HPV vaccine was also introduced in 2008 across 125 municipalities with a low human development index, representing approximately 5% of the population and associated with the highest rates of CC. Quadrivalent HPV vaccine was provided through mobile health clinics to girls aged 12 to 16, adhering to a three-dose schedule. Mexico approved a nationwide expansion of its HPV vaccination program in 2011 to encompass school-based vaccination for all girls aged 9 years [26]. In Brazil, the HPV vaccine was introduced in 2014 for girls aged 9 to 14 and extended to boys in the same age group in 2017, as health center-based following a two-dose schedule [27].

Although most national immunization programs have traditionally focused on vaccinating girls, there is increasing recognition that extending vaccination to boys is essential to provide direct protection against HPV-related cancers in men and to enhance herd immunity [10]. Initial resistance to this policy was largely due to concerns about cost-effectiveness and the assumption that high coverage among girls would be sufficient. However, growing evidence of HPV-associated cancers in men and indirect benefits from herd protection have led many high- and middle-income countries to adopt gender-neutral vaccination strategies. In contrast, several LATAM countries—including Costa Rica, the Dominican Republic, Guyana, Honduras, Mexico, and Nicaragua—have yet to include boys in their HPV vaccination programs (Table 1).

Since its implementation, an interesting pattern has emerged in countries with available HPV vaccine data: adherence to the second dose is consistently lower than the first (Table 2) [28]. In response, WHO revised its recommendations to endorse a single-dose option for individuals aged 9–20, based on evidence demonstrating similar efficacy in preventing HPV infections and cervical precancerous lesions. Two doses are still recommended for those aged 21 years or older, and at least two, preferably three, doses are recommended for those who are immunocompromised [29]. This strategy aims to improve adherence and accessibility and has been implemented in some countries. In Brazil, the Ministry of Health changed the immunization regimen for girls and boys aged 9–14 years old from a two-dose to a single-dose scheme in 2024 [30].

## 5. Current Landscape of HPV Vaccination in Latin America

Mortality rates of CC are three times higher in LATAM and the Caribbean than in North America, highlighting severe health inequalities and the urgent need for interventions to reduce disease burden [1]. According to the WHO, among the 194 countries worldwide, 144 have included the HPV vaccine in their national immunization programs, while 48 have not. In America, only Venezuela, Haiti, and Cuba have not included the HPV vaccine in their national immunization programs [31]. In accordance with the usual recommendations for HPV vaccination (ages 9–14 for males and females; up to 45 years for immunosuppressed patients regardless of gender), an analysis was conducted on vaccination coverage across LATAM and within each of the 19 countries that comprise this region, considering the period between 2010 and 2023 (Table 1). The analysis showed that vaccination coverage has been increasing in both sexes; however, the uptake of the first vaccine dose has diminished in the years following its incorporation, which has been marked by significant variability in national immunization calendars, as well as a tendency in declining of adherence for the second dose across all countries with available vaccine data has been observed [28].

The HPV vaccination delivery strategy differs across countries. For instance, Brazil adopted a facility-based vaccination approach, while Bolivia, Peru, and Chile implemented a school-based vaccination approach. The coverage of the first dose of the HPV vaccine was reported by the WHO in 2023 and varies among LATAM countries (Table 2). Brazil, Peru, Chile, and Ecuador have coverage rates greater than 70%. On the other hand, Paraguay and Guyana have coverage rates of less than 45%. To date, no LATAM country has achieved the WHO target of 90% of girls fully vaccinated against HPV by age 15 [13].

Regarding CC screening in the Americas region, 78% of women aged 25–65 years have been screened at least once in their lifetime. In South America, this rate is 68%, also falling short of the target of 70% of women screened with a high-performance test by the age of 35. In Brazil, a country of continental dimensions, only 58% of women have ever undergone a screening test in their lifetime [32].

Regarding the impact of HPV vaccination on cervical cancer and high-grade cervical intraepithelial neoplasia in LATAM, most previous evidence in LMICs has relied on mathematical models. However, real-world data from Brazil demonstrated that the national HPV vaccination programme was associated with a 58% reduction in cervical cancer and a 67% reduction in CIN3 among women aged 20–24 years. These findings, consistent with results from high-income countries, confirm that HPV vaccination can significantly reduce disease burden even in resource-limited settings, although complete elimination will still require integration with robust screening programmes [33].

## 6. Barriers and Challenges

The lack of knowledge regarding the HPV vaccine remains a significant barrier to widespread immunization. A cross-sectional study conducted among Brazilian teenagers revealed that 45.54% of the participants were unaware of the HPV vaccine, with higher rates observed among those who had initiated sexual activity, attended public schools and resided in the Northeast Region [34]. These findings underscore the urgent need for expanded educational initiatives to raise awareness and encourage vaccination.

In Brazil, despite the implementation of a national HPV vaccination program in 2014, the coverage rate remains below the WHO target of 90%. This underscores the urgent need for targeted interventions to improve vaccine uptake. Additionally, there are regional disparities in vaccination coverage, with lower adherence in the North and Northeast regions, possibly due to limited healthcare infrastructure, lower socioeconomic status, and reduced access to vaccination services [28,34].

Another major challenge is the cultural stigmas associated with HPV vaccination. Since HPV is a sexually transmitted infection (STI), some parents fear that vaccinating their children may encourage early sexual initiation and increased sexual activity. Such misconceptions negatively affect parental acceptance and trust in the vaccine [35]. A study conducted among healthcare providers in Minnesota found that 79% of parents believed their child was not sexually active, and 63% assumed their child would not contract HPV-related diseases [36]. This highlights the powerful role of misinformation in parental decision-making, ultimately reducing vaccine uptake.

Conservative religious perspectives, combined with the characterization of HPV as a sexually transmitted infection, contribute to reinforcing cultural taboos that limit communication and education about HPV. Nevertheless, current evidence indicates that religiosity is not a consistent barrier to vaccination. Studies involving Catholic, Evangelical, Black, and Hispanic parents in the United States have shown no direct association between religious attendance or salience and HPV vaccine uptake, suggesting that factors such as perceived benefits, barriers, and physician recommendation exert greater influence on decision-making [37,38,39,40]. However, in more conservative environments, such as Christian universities, stronger religious beliefs regarding sexual abstinence have been associated with higher levels of hesitancy [39]. Importantly, weak or delayed physician recommendations appear to amplify parental doubts regardless of religious background, underscoring that healthcare communication, rather than religiosity itself, is the critical determinant of vaccine acceptance [40].

Furthermore, nonadherence to the HPV vaccination 3-dose regimen remains a critical challenge. In a California-based study involving 34,193 young women who initiated the quadrivalent HPV vaccine, the completion rate was 41.9% among those aged 9 to 17 years and 47.1% among those aged 18 to 26 years [41]. Additionally, a retrospective cohort study including 378,484 females aged 9 to 26 years in the United States reported that only 29.4% completed the full HPV vaccination series [42]. In Brazil, the completion rate is also suboptimal, particularly among boys. Contributing factors include vaccine hesitancy, logistical barriers, and lack of reinforcement from healthcare providers [43].

The COVID-19 pandemic further exacerbated the decline in HPV vaccination rates due to the disruption of routine immunization programs and a shift in healthcare priorities. A significant drop in HPV vaccine coverage was reported in multiple countries, where catch-up vaccination efforts are now crucial to recovering lost immunization rates and preventing future HPV-related diseases [44].

In addition, social media has emerged as a critical factor influencing HPV vaccine perceptions. While these platforms can improve awareness, they also amplify misinformation, particularly around safety and sexual behavior, which contributes to hesitancy [45]. Anti-vaccine content often receives higher engagement than supportive messages, further extending its reach and impact. Nonetheless, evaluations of targeted campaigns demonstrated that combining clear cancer-prevention messaging with empathetic language and peer-to-peer dialogue can effectively counter misinformation and promote engagement [46]. More recently, studies using large language models to analyze over 650,000 HPV-related tweets revealed that dominant concerns include adverse effects, personal anecdotes of vaccine injury, and debates on mandates and parental consent, emphasizing the evolving nature of online discourse and the need for dynamic monitoring to inform public health strategies [47].

Cost is another substantial barrier, particularly in LMICs, where the HPV vaccine is one of the most expensive childhood immunizations globally available. The cost exceeds $300 per full series. This financial barrier severely limits access for vulnerable populations and constrains national immunization program coverage [48].

## 7. Potential Solutions to Overcome Barriers and Future Perspectives

Enhancing knowledge and awareness is a proven strategy for increasing HPV vaccine adoption. Countries such as Australia, Mexico, and Peru have successfully surpassed the WHO-recommended 80% coverage rate by implementing targeted social marketing campaigns that effectively engaged their populations [49,50]. Additionally, the use of online environments and social media campaigns has proven effective in increasing vaccine awareness and engagement, particularly among younger populations who are more active on these platforms [51].

The role of healthcare providers is also crucial in addressing safety concerns and dispelling misconceptions surrounding HPV vaccination [52]. Additionally, school-based vaccination programs serve as vital safety-net institutions that significantly contribute to vaccination rates. A randomized trial conducted in 91 French municipalities demonstrated that in-school HPV vaccination increased coverage by 5.50 percentage points (95% CI, 3.13–7.88 percentage points) compared to conventional approaches [53].

Furthermore, simplifying the vaccination regimen could improve adherence and reduce costs. A randomized trial in India involving 17,729 women evaluated different dose schemes of the quadrivalent HPV vaccine and concluded that a single dose is immunogenic and provides long-lasting protection against HPV 16 and 18 infections, comparable to the three- and two-dose schedules [54]. Based on this evidence, the WHO Strategic Advisory Group of Experts on Immunization (SAGE) revised its guidelines to endorse a single-dose option for individuals aged 9–20 [29]. This approach facilitates broader vaccine coverage by addressing adherence issues, reducing financial burdens, and enabling more patients to receive the vaccine.

**Figure 2:**
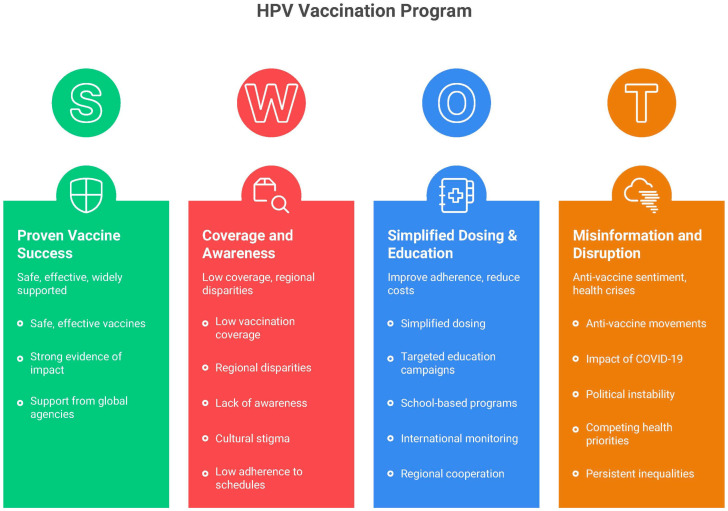
SWOT analysis of HPV vaccination in Latin America.

Additionally, policy interventions could play a critical role in increasing HPV vaccine uptake. Some countries, such as the United Kingdom and Australia, have implemented mandatory school-based vaccination programs, which have resulted in higher coverage rates and a significant decline in HPV-related diseases [55,56]. In LMICs, introducing financial incentives or expanding publicly funded vaccination could help overcome cost barriers and expand access to vulnerable populations [28]. A comprehensive overview of strengths, weaknesses, opportunities, and threats related to HPV vaccination in Latin America is exemplified in Figure 2, providing a structured framework to guide these policy decisions.

In this context, monitoring and evaluating the real-world impact of HPV vaccination is essential to guide and optimize prevention efforts. To address this need, the International Agency for Research on Cancer (IARC) launched in June 2025 the Center of Excellence for Monitoring HPV Vaccination Impact (CHRONOS), the first global repository of standardized data aimed at tracking outcomes, particularly in LMICs [9]. This initiative is especially relevant for regions such as LATAM, where the paucity of official data and the absence of robust registries and standardized systems continue to limit accurate evaluation of long-term impact and perpetuate gaps in CC prevention.

The combined use of high-coverage HPV vaccination and optimized cervical screening supports Australia’s strategy to fight cervical cancer. The National HPV Vaccination Program began in 2007 for girls and was extended to boys in 2013; approximately 80% of girls under 15 years achieved full coverage with the 4vHPV, with subsequent declines in HPV prevalence, high-grade cervical lesions and genital warts and documented herd effects [57]. Because cervical carcinogenesis has a long latency, older cohorts continue to rely primarily on screening. In 2017 the National Cervical Screening Program replaced two-year cytology with five-year primary HPV screening, increasing sensitivity and enabling earlier detection of prevalent lesions [58,59]. The planned rollout of the 9vHPV is expected to extend protection against additional oncogenic types and could prevent up to 90% of cervical cancers. Sustained high vaccination uptake combined with accessible, evidence-based screening provides an integrated, scalable framework for reducing cervical cancer burden [60].

Looking ahead, HPV vaccination in LATAM requires coordinated and innovative approaches to ensure sustained progress toward elimination goals. The PAHO Revolving Fund for Vaccine Procurement exemplifies how regional pooled purchasing mechanisms can successfully negotiate lower prices and ensure equitable access to HPV vaccines, providing a model that could be further expanded through frameworks such as MERCOSUR [61]. Public–private partnerships are also expected to become increasingly relevant, particularly for expanding access among vulnerable populations and ensuring the sustainability of national immunization programs. Collaborations with international organizations, philanthropic foundations, medical societies, advocacy groups—such as the Brazilian Gynecologic Tumor Group (EVA)—and the private sector could secure long-term financing, strengthen supply chains, and enhance accountability for expanding HPV vaccination [62]. Finally, advances in artificial intelligence and data science offer unique opportunities for real-time monitoring of HPV vaccination coverage and impact, with tools such as large language models enabling the detection of emerging misinformation and supporting evidence-based interventions tailored to population needs [46].

By implementing these strategies—education, healthcare provider advocacy, school-based programs, digital engagement, policy interventions, and simplified dosing regimens—it is possible to overcome barriers and significantly increase HPV vaccination coverage, ultimately contributing to the reduction of HPV-related diseases and CC incidence globally.

## 8. Conclusions

Despite being a crucial advancement in public health, HPV vaccination in LATAM faces significant challenges that limit its effectiveness. Although most countries have included the vaccine in their national immunization programs, coverage targets are largely unmet, particularly in regions where the vaccine is not yet widely available. Barriers such as limited public awareness, cultural stigma, safety concerns, logistical difficulties, and poor integration with healthcare services contribute to persistently low adherence—especially regarding completion of multidose regimens. To improve HPV vaccination efforts, strategies must be tailored to local contexts. Promising approaches include engaging healthcare providers, integrating vaccination into schools, and adopting a single-dose schedule. These approaches, along with increased vaccine access, enhanced public awareness, and consistent program monitoring, are essential for achieving higher coverage and advancing CC elimination in LATAM.

## Figures and Tables

**Table 1:** Vaccination Data in LATAM According to WHO.

Country	Year of HPV Vaccine Implementation	Target Sex	Schedule (9–14 Years Old)	HPV Vaccination Program Coverage, First Dose, Females (2023)	HPV Vaccination Program Coverage, Last Dose, Females (2023)	HPV Vaccine Primary Delivery Strategy
**Argentina**	2011	F/M	1	63%	36%	MX
**Bolivia**	2017	F	1	67%	67%	SB
**Brazil**	2014	F/M	1	87%	73%	FB
**Chile**	2014	F/M	2	88%	83%	SB
**Colombia**	2012	F/M	1	52%	52%	SB
**Costa Rica**	2019	F	2	77%	66%	SB
**Dominican Republic**	2017	F	1	66%	66%	FB
**Ecuador**	2014	F/M	2	79%	50%	SB
**El Salvador**	2020	F/M	1	76%	36%	SB
**Guatemala**	2018	F/M	1	48%	48%	SB
**Guyana**	2011	F/M	1	40%	40%	SB
**Honduras**	2016	F	1	81%	61%	SB
**Mexico**	2012	F	1	62%	62%	SB
**Nicaragua**	2023	F	2	78%	N/A	SB
**Panama**	2008	F/M	2	79%	66%	MB
**Paraguay**	2013	F/M	1	44%	22%	MB
**Peru**	2015	F/M	1	74%	74%	SB
**Suriname**	2013	F/M	1	N/A	N/A	SB
**Uruguay**	2013	F/M	2	67%	41%	FB

F = Female M = Male NA = Not available SB = School-based FB = Facility-based MX = mixed. Data gathered from World Health Organization report’s—Human papillomavirus (HPV) vaccination coverage [13].

**Table 2:** Vaccination coverage in immunization programs in Latin America between 2010 and 2023 (%).

	2023	2022	2021	2020	2019	2018	2017	2016	2015	2014	2013	2012	2011	2010
First dose—females	68	69	53	57	60	66	69	62	60	64	38	32	17	11
First dose—males	43	39	39	38	39	38	27	15	14	11	9	5	3	0
Last dose—females	55	54	39	39	54	53	58	51	43	44	29	24	10	6
Last dose—males	33	29	28	26	26	28	13	9	7	4	3	2	1	0

Data gathered from World Health Organization report’s—Human papillomavirus (HPV) vaccination coverage [13].

## Data Availability

No new data were created or analyzed in this study.
